# The development of the Police Practices Scale: Understanding policing approaches towards street-based female sex workers in a U.S. City

**DOI:** 10.1371/journal.pone.0227809

**Published:** 2020-01-24

**Authors:** Katherine H. A. Footer, Ju Nyeong Park, Saba Rouhani, Noya Galai, Bradley E. Silberzahn, Steven Huettner, Sean T. Allen, Susan G. Sherman

**Affiliations:** 1 Department of Health, Behavior and Society, Johns Hopkins Bloomberg School of Public, Baltimore, MD, United States of America; 2 Department of Epidemiology, Johns Hopkins Bloomberg School of Public Health, Baltimore, MD, United States of America; 3 Department of Statistics, University of Haifa, Mt Carmel, Israel; 4 Johns Hopkins School of Medicine, Baltimore, MD, United States of America; University of North Carolina at Chapel Hill, UNITED STATES

## Abstract

Policing is an important structural determinant of HIV and other health risks faced by vulnerable populations, including people who sell sex and use drugs, though the role of routine police encounters is not well understood. Given the influence of policing on the risk environment of these groups, methods of measuring the aggregate impact of routine policing practices are urgently required. We developed and validated a novel, brief scale to measure police patrol practices (Police Practices Scale, PPS) among 250 street-based female sex workers (FSW) in Baltimore, Maryland, an urban setting with high levels of illegal drug activity. PPS items were developed from existing theory and ethnography with police and their encounters with FSW, and measured frequency of recent (past 3 months) police encounters. The 6-item scale was developed using exploratory factor analysis after examining the properties of the original 11 items. Confirmatory factor analysis was used to model the factor structure. A 2-factor model emerged, with law enforcement PPS items and police assistance PPS items loading on separate factors. Linear regression models were used to explore the relative distribution of these police encounters among FSW by modeling association with key socio-demographic and behavioral characteristics of the sample. Higher exposure to policing was observed among FSW who were homeless (β = 0.71, p = 0.037), in daily sex work (β = 1.32, p = 0.026), arrested in the past 12 months (β = 1.44, p<0.001) or injecting drugs in the past 3 months (β = 1.04, p<0.001). The PPS provides an important and novel contribution in measuring aggregate exposure to routine policing, though further validation is required. This scale could be used to evaluate the impact of policing on vulnerable populations’ health outcomes, including HIV risk.

## Introduction

Considerable progress has been made in demonstrating the role of social and structural factors in determining risk trajectories in public health. The combination of legislative, economic, social and physical constraints exogenous to the individual are now understood to shape a *‘risk environment*,*’* that is critical in determining health outcomes among vulnerable populations.[1] In particular, the risk environment has provided an important organizing framework to elucidate important structural factors impacting health outcomes among populations involved in drug use and sex work, including transmission of HIV and other bloodborne infections, drug overdose, and experiencing violence.[2–6]

The role of criminalization and policing as important structural risk factors is increasingly recognized in social epidemiological literature[7,8]. Structural violence refers to a form of violence embedded within social structures at multiple levels, wherein lack of power translates to tangible disadvantage or risk[9]. The law in this context can be conceptualized as an ecological risk factor[10], whose translation from *‘the books’* to *‘the street’* constitutes a form of structural violence at the macro and micro levels. At the macro level *(‘the books’*), sex work and drug use are largely criminalized in the United States, with highly punitive legislation manifesting as both sanctioned and unsanctioned forms of discretionary policing that influence harms related to drug use and sex work.[10] *At the micro level (‘the street’)*, *police practices including arrest*, *stop and search*, *moving women along impact health behaviors (e*.*g*., *client screening*, *condom use) and exposures (e*.*g*., *violence) among vulnerable street-based populations*, *such as female sex workers (FSW) and people who use drugs (PWUD)[11], and oppressive and coercive practices such as harassment*, *sex in exchange for no arrest are normalized in their day to day lives.[12]* At the micro level *(‘the street’*), police practices impact health behaviors and exposures among vulnerable street-based populations, such as female sex workers (FSW) and people who use drugs (PWUD), and oppressive and coercive practices are normalized in their day to day lives.

Globally, studies have emphasized different types of abusive (e.g., sexual and physical assault) and enforcement based (e.g., police crackdowns, arrest, syringe confiscation) policing practices as contributors to risk behaviors and harmful experiences, including inconsistent condom use[13,14], needle sharing and unwillingness to carry syringes[15,16], drug use[17,18], access to services[19,20], experiences of violence[21–23] and depression.[24] A recent systematic review exploring policing practices as a structural determinant of HIV and other sexually transmitted infection (STI) amongst FSW found arrest, syringe confiscation, police extortion and sexual coercion to be positively associated with infection.[25] Similarly, studies among PWUD have pointed to the fear of police interaction as a salient barrier to accessing harm reduction resources to reduce risk of infectious disease transmission such as HIV and hepatitis C, abscesses and wound infections, and resources to prevent and reverse overdose.[4,26,27]

To date, emphasis in the literature has understandably been on egregious practices, particularly among FSW, with a focus on physical and sexual assault. Examples of efforts to better translate the interacting typologies (policies, social, economic, and physical) and levels (micro, macro) comprising the ‘risk environment’ framework into more robust quantitative scales are lacking[6], particularly when the exposure of interest is aggregate. The majority of literature reduces the broad range of police interactions to a single measure, usually arrest or violence, to define exposure to policing, and studies with more nuanced definitions lack generalizability and comparative value due to the different items measured[25]. Given the growing evidence of the impacts of routine patrol police practices (e.g., stop and search) on mental health outcomes in the wider population[28–30], there is a need to understand how these may impact uniquely vulnerable groups like FWS and PWUD. Analytic tools are needed to better characterize the potentially insidious effect of frequent interactions between police and vulnerable communities, which when considered individually would not be expected to incur harm.

To address this gap, this paper describes the development and initial validation of a novel and brief scale that measures routine police patrol practices experienced by FSW, focusing on the micro environment of the street and in the context of policing street-based FSW, a large proportion of whom are also PWUD. In creating this scale, we hypothesized that FSW’s aggregate experiences of exposure to day to day policing practices, whether legal or extrajudicial, collectively impact women’s risk environment and likelihood of experiencing health related harms. We also explored the basic socio-demographic and behavioral factors associated with increased exposure to policing as measured by the Police Practices Scale (PPS). The study aims to provide a scale for subsequent validation and use in the North American context and adaption elsewhere, allowing for the more systematic examination of the impact of existing police patrol practices among key populations and assisting in both future intervention design and implementation.

## Methods

### Study population

The Sex workers And Police Promoting Health In Risky Environments (SAPPHIRE) study is a prospective cohort study examining the role of the police in influencing the HIV/STI risk environment of street-based FSW in Baltimore. The sample was comprised of 250 cisgender FSW and 62 transgender FSW who were enrolled between 2016–2017 and followed up for 12 months. A detailed description of the recruitment methods, study procedures and participant characteristics are published elsewhere.[31] Participants were recruited through targeted sampling methods at street-based locations across Baltimore city.[32] To be eligible to participate, women had to be aged 15 years or above, self-identify as female, engaged in street-based sex work at least 3 times in the past 3 months, and provide informed consent. During the baseline visit, participants completed a 50-minute interviewer-administered computer assisted personal interview (CAPI) on the study van and underwent HIV and STI testing. Participants were compensated with a pre-paid $70 VISA gift card for completing the baseline visit. The study was approved by the Johns Hopkins Bloomberg School of Public Health Institutional Review Board.

Given the small sample size of transgender participants (women assigned male at birth; n = 62), analyses were restricted to the cisgender cohort. However, descriptive data on the use of this scale among transgender FSW (frequency of exposure to scale components and overall internal consistency of the PPS) are shown in supplementary tables.

### Development of the PPS scale

The police encounters measured through the Patrol Practices Scale (PPS) were grounded in existing ecological[10] and risk environment frameworks as well as ongoing ethnography.[1] Drawing on these frameworks we explored policing as a micro factor impacting on the physical risk environment of the ‘street’ and explored how criminalization and surrounding policies translated to street-level routine police patrol practices towards FSW. We conceptualized ‘routine police patrol practices’ as those that are standard practice from a police perspective (e.g., filed interview, stop and search). A systematic review of the quantitative FSW literature was used to identify gaps and opportunities in the development of police measurement items.[25] To develop a range of police measurement items, we augmented findings from the literature with preliminary results from an ongoing police ethnography in Baltimore City, that observed enforcement practices with FSW in the context of the SAPPHIRE study.[25] Measurement items were discussed with members of the SAPPHIRE community advisory board (CAB), comprised of former and current sex workers.

**Fig 1:**
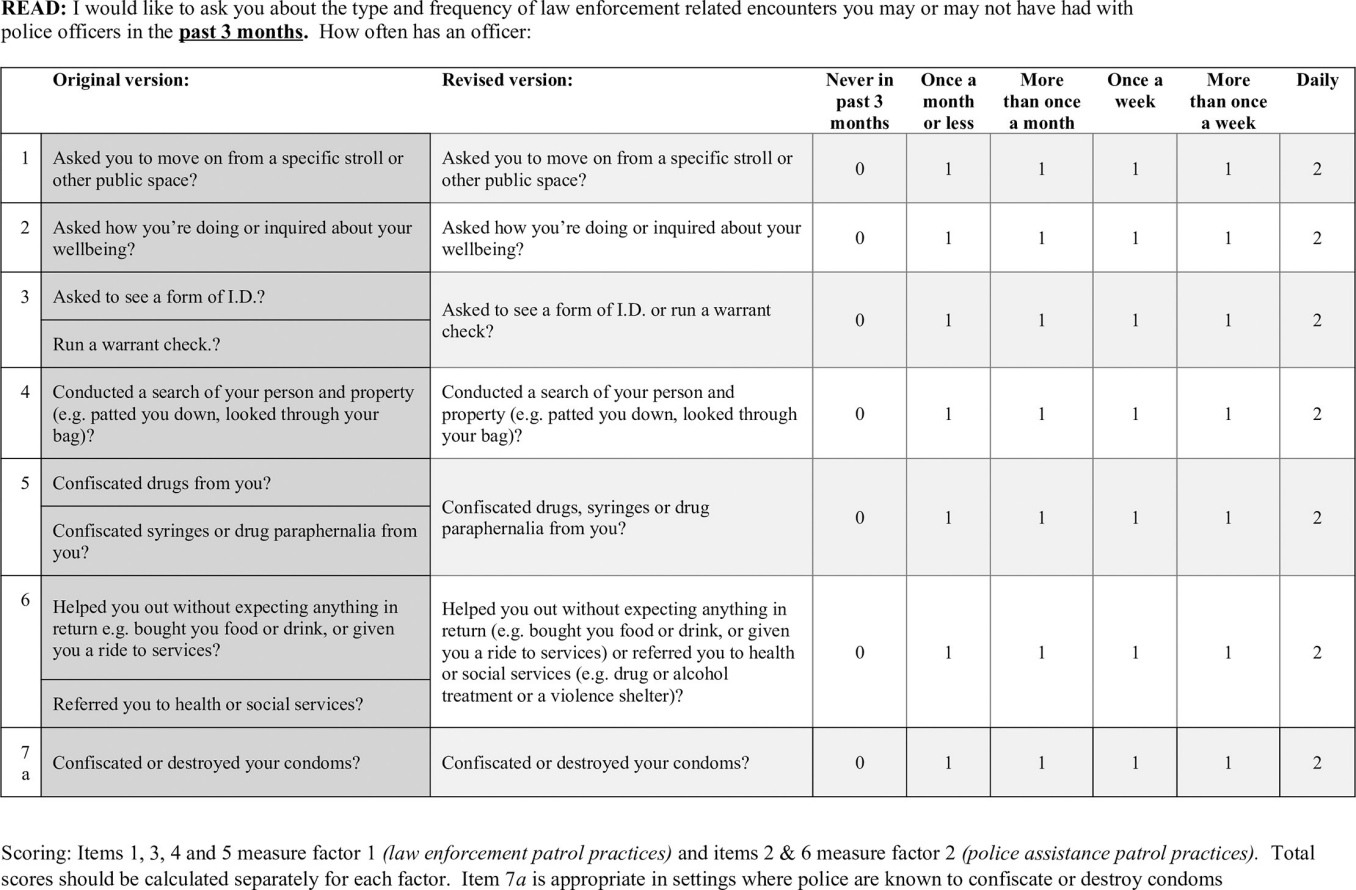
Police Patrol Practices Scale (PPS). Items included in the box were read to participants after the phrase *“*: *I would like to ask you about the type and frequency of law enforcement related encounters you may or may not have had with police officers in the* ***past 3 months***. *How often has an officer…[insert item]*?*”* Scoring is shown for original and revised scales. Items 1, 3, 4 and 5 measure factor 1 *(law enforcement patrol practices)* and items 2 & 6 measure factor 2 *(police assistance patrol practices)*. Total scores should be calculated separately for each factor. Item 7*a* is appropriate in settings where police are known to confiscate or destroy condoms.

For each PPS item, we ascertained lifetime prevalence as well as recent (past three month) frequency. For each type of routine police patrol practice, respondents were asked if a police officer had ever engaged in the listed behavior, and if so, how often it happened in the past 3 months. The response options, which were displayed on a flashcard and read aloud by the interviewer, were as follows: daily, more than once a week, once a week, more than once a month, once a month or less, never. The exact wording of each item is shown in Fig 1. Two items, ‘asked to see a form of identification’ and ‘warrant check’ represent common patrol practices in this context and involve checking the person has a form of legal I.D. (e.g., driving license) and using the identifying information to check the police databases for ant open warrant, which is a warrant for arrest issued by a judge in lieu of a failed court appearance. The 11^th^ item (issuing a contact sheet for stopping a citizen) was not included in analysis due to ethnographic findings highlighting that it was highly correlated with these other items.

Prior to the PPS, respondents were also asked a broad question on the frequency of any police interactions in their lifetime using: “How often do police officers say something to you? It doesn't matter whether or not it resulted in the officer taking any official action.” Respondents who answered “never” were asked a follow-up question: “Just to confirm, you are saying that a police officer has never said anything to you in your life and you have never been arrested or locked up in jail, prison or a correctional facility?” Respondents who confirmed that police had never spoken to them in their life (n = 16) were not asked the PPS however their responses to the PPS variables were coded as “never” in order to retain them in the analysis.

### Socio-demographic and behavioral covariates

At baseline, we captured data on age, highest level of education (dichotomized for analysis: did not complete high school vs. completed high school/GED/higher education) and homelessness (“have you been homeless in the past 3 months”). The survey also included items on race/ethnicity, which was collapsed into a three-level variable for regression analysis: Non-Hispanic White vs. Non-Hispanic Black vs. Hispanic, Multiracial or Other.

Responses to length in street-based sex work was captured using a range of frequencies and collapsed into three groups for analysis (≤1 year vs. >1 to 5 years vs. >5 years). Frequency of street-based sex work in the past 3 months (daily vs. ≤ weekly vs. ≤ monthly), arrest (past 12 months) and sources of income in the past 3 months (consisted of a range of response options, one of which was “Selling, touting, or steering drugs”) were also measured.

Daily heroin or cocaine use (yes vs. no) was indicated by selection of “daily” for any of the following drug use frequency items: injected heroin and cocaine together; snorted or smoked heroin and cocaine together; smoked crack; sniff or snorted cocaine by itself; injected cocaine by itself; injected heroin by itself; sniffed or snorted heroin by itself.

### Scale refinement and evaluation

The frequencies and polychoric correlations of the original PPS items were examined. The condom confiscation item was not included in factor analysis as it was a rare event in our sample (4%). Variables that were strongly correlated (rho≥0.8) were collapsed for factor analysis (i.e. we collapsed items 3 & 4; items 7 & 8; items 9 & 10). We also collapsed their response options due to sparseness of data (2 = daily or weekly vs. 1 = < weekly vs. 0 = never in the past 3 months). The final scale consisted of 6-items with scores ranging from 0–12.

Parallel analysis was conducted to determine the number of factors to extract. Exploratory Factor Analysis (EFA) was used to examine the factor loadings and uniqueness of each item. A sensitivity analysis was also conducted to ascertain the factor structure of the original 11-item scale without collapsed indicators. The multivariate normality assumption was tested using an omnibus test for multivariate normality [33]. Total scores and internal consistency (Cronbach’s alpha) statistics were calculated for the overall PPS scale as well as each factor.

Confirmatory Factor Analysis (CFA) was conducted using Mplus 7.0 (Muthén & Muthén, 1998–2012). Weighted Least Squares Means and Variance Adjusted (WLSMV) estimation, which is based on the polychoric correlation matrix of latent continuous response variables, was used to conduct the CFA. A one factor model and a two-factor model (law enforcement patrol practices: drugs, routine, move along and search; police assistance patrol practices: ask how and assistance) were run. Factor variances were set to 1 and factors were oblique. The factor loadings and standard errors were examined. Model fit was assessed using the Root Mean Square Error of Approximation (RMSEA) [34], the Comparative Fit Index (CFI) [35], and the Tucker-Lewis Index (TLI) [36]. The quality of model fit was assessed using established cut-offs: RMSEA<0.8 reflected adequate fit; CFI>0.95 and TLI>0.95 reflected excellent fit.

Bivariate linear regression models were used to explore the associations between *a priori* determined covariates (key socio-demographic and behavioral characteristics) and the total PSS score as well as each emergent factor. All descriptive, exploratory analyses and regression analyses were conducted in Stata/SE 14.2 (College Station, Texas).

## Results

### Sample characteristics

The average age of the sample was 36 years (SD = 9) and ranged from 18 to 61 years. The sample was 66% White, 23% Black and 11% Hispanic/multiracial/other. The majority of the sample did not complete high school (52%) and experienced homelessness in the past 3 months (62%). About half (52%) had been in street-based sex work for more than 5 years, and most worked daily (66%). Prior year arrest experiences were common (47%). The majority injected drugs in the past 3 months (71%), predominantly heroin and cocaine. Almost a quarter (23%) sold or supplied drugs.

### Patrol Practices Scale

The frequencies of the PPS items are shown in Table 1. The most frequent recent encounters were being asked to move along (67%), being asked for I.D. (57%) and having one’s warrant checked (57%). The least frequent encounters were having condoms (4%) or drugs (16%) confiscated, receiving direct assistance (17%) and being referred to services (12%). The polychoric correlations between PPS items and sex work frequency are displayed in Table 2. The highest correlations (rho≥0.8) were observed between items 3 & 4, items 7 & 8 and items 9 & 10.

**Table 1:** Police interactions measured using the Patrol Practices Scale (PPS) items among female sex workers (N = 250) in Baltimore, Maryland.

Item no.		Never in past 3 months	Once a month or less	More than once a month	Once a week	More than once a week	Daily
1	Asked you to move on from a specific stroll or other public space.	81 (32.5)	72 (28.9)	21 (8.4)	24 (9.6)	33 (13.3)	18 (7.2)
2	Asked how you’re doing or inquired about your wellbeing.	137 (54.8)	61 (24.4)	19 (7.6)	14 (5.6)	12 (4.8)	7 (2.8)
3	Asked to see a form of I.D.	106 (42.6)	79 (31.7)	20 (8.0)	16 (6.4)	20 (8.0)	8 (3.2)
4	Run a warrant check.	107 (43.0)	80 (32.1)	25 (10.0)	14 (5.6)	17 (6.8)	6 (2.4)
5	Conducted a search of your person and property (e.g. patted you down, looked through your bag).	151 (60.4)	65 (26.0)	14 (5.6)	10 (4.0)	7 (2.8)	3 (1.2)
6	Confiscated or destroyed your condoms.	240 (96.4)	7 (2.8)	1 (0.4)	0 (0.0)	1 (0.4)	0 (0.0)
7	Confiscated drugs from you.	209 (83.6)	31 (12.4)	3 (1.2)	2 (0.8)	4 (1.6)	1 (0.4)
8	Confiscated syringes or drug paraphernalia from you.	199 (79.9)	40 (16.1)	4 (1.6)	2 (0.8)	3 (1.2)	1 (0.4)
9	Helped you out without expecting anything in return e.g. bought you food or drink, or given you a ride to services.	207 (82.8)	35 (14.0)	3 (1.2)	3 (1.2)	1 (0.4)	1 (0.4)
10	Referred you to health or social services e.g. drug or alcohol treatment, or a violence shelter.	221 (88.4)	24 (9.6)	1 (0.4)	1 (0.4)	2 (0.8)	1 (0.4)

Note: For factor analysis, the response options were collapsed into three groups (never, <daily and daily)

**Table 2:** Polychoric correlation matrix of Patrol Practices Scale (PPS) items.

Item no.		Move along	Ask how	I.D.	Warrant check	Search	Condoms	Drugs	Drug para	Helped	Referred
1	Move along	1									
2	Wellness check	.23	1								
3	I.D.	.68	.33	1							
4	Warrant check	.66	.29	.83	1						
5	Search	.64	.18	.66	.71	1					
6	Condoms	.25	.41	.21	.22	.24	1				
7	Drugs	.36	.30	.37	.40	.56	.33	1			
8	Drug para	.54	.34	.51	.54	.71	.56	.79	1		
9	Helped	.12	.56	.04	.02	.09	.33	.32	.18	1	
10	Referred	.03	.44	.01	.14	.25	.35	.26	.21	.79	1

Note: Highlighted boxes indicate correlation coefficients greater than .6.

As shown in Table 3, exploratory factor analysis of the final 6-item scale yielded a two-factor model. Law enforcement patrol practices consisted of four items (asked to move along, routine warrant or ID check, conducted a search, confiscated drugs or drug paraphernalia) and police assistance patrol practices consisted of two items (asked about wellbeing, provided assistance). The sensitivity analysis of the original 11-item scale (i.e., without collapsing items) also supported a two-factor model (data not shown). The mean score of the final scale was 2.8 (SD = 2.0) and scores ranged from 0 to 11. The mean (SD) and range for law enforcement patrol practices were 2.1 (SD = 1.6) and 0.7 (SD = 0.8) respectively. We rejected the null hypothesis of multivariate normality of the PPS (p = 0.01). The internal consistency of the overall PSS (α = 0.73) and law enforcement patrol practices items (α = 0.79) was acceptable, and police assistance patrol practices was low (α = 0.53).

**Table 3:** Exploratory Factor Analysis of the Police Patrol Practices Scale (PPS) among FSW in Baltimore, Maryland (N = 250).

		Factor loadings			
no.	Item	Analytic item	Law enforcement patrol practices	Police assistance patrol practices	Unique-ness
1	Asked you to move on from a specific stroll or other public space.	*Move along*	**0.814**	-0.120	0.374
2	Asked how you’re doing or inquired about your wellbeing.	*Ask how*	0.067	**0.757**	0.396
3	Asked to see a form of I.D.	*Routine*	**0.824**	-0.036	0.336
4	Run a warrant check.				
5	Conducted a search of your person and property (e.g. patted you down, looked through your bag).	*Search*	**0.819**	-0.011	0.334
6	Confiscated or destroyed your condoms.	*--*	--	--	--
7	Confiscated drugs from you.	*Drugs*	**0.574**	0.301	0.490
8	Confiscated syringes or drug paraphernalia from you.				
9	Helped you out without expecting anything in return e.g. bought you food or drink, or given you a ride to services.	*Assistance*	-0.126	**0.858**	0.306
10	Referred you to health or social services				
*Cronbach’s α*					
	Full scale (9-items; excluding item 6)				0.77
	Final scale (6-items; collapsed items 3 & 4; items 7 & 8; items 9 & 10)				0.73
	Law enforcement patrol practices (4-items)				0.79
	Police assistance patrol practices (2-items)				0.53

### Confirmatory Factor Analysis

**Fig 2:**
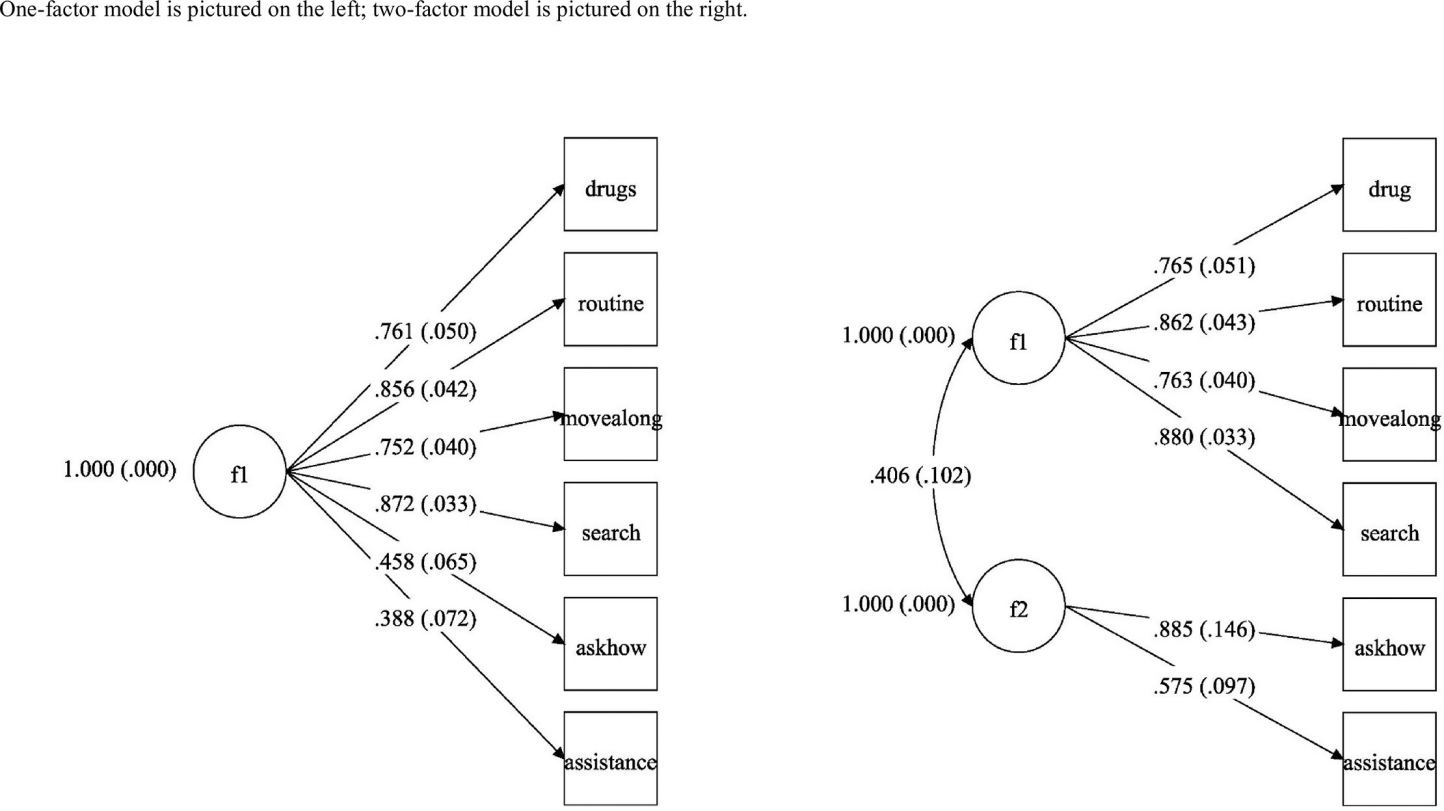
Factor loadings, standard errors and factor covariance from confirmatory factor analysis of the 6-item PPS. One-factor model is pictured on the left; two-factor model is pictured on the right.

Factor loadings for the one-factor and two-factor CFA models are shown in Table 4. The one-factor model did not meet established model fit cut-offs (RMSEA = 0.181; CFI = 0.911; TLI = 0.852). However, the two-factor model exhibited marginally adequate fit according to the RMSEA (RMSEA = 1.01) and had excellent fit according to results of the CFI and TLI (CFI = 0.976; TLI = 0.954). Factor loadings ranged from 0.575–0.880. Factor loadings for the two-factor model were stronger than in the one-factor model. All factor loadings were statistically significant (*p*<0.001). The factor loadings, standard errors and factor covariance are displayed in Fig 2. As expected, the two factors had a positive covariance.

**Table 4:** Item factor loadings and significance of the Confirmatory Factor Analysis models.

	One factor model		Two factor model		
Item	*Law enforcement patrol practices*	p value	*Law enforcement patrol practices* *(Factor 1)*	*Police assistance patrol practices* *(Factor 2)*	p value
Drugs	0.761	**<0.001**	0.765		**<0.001**
Routine	0.856	**<0.001**	0.862		**<0.001**
Move along	0.752	**<0.001**	0.763		**<0.001**
Search	0.872	**<0.001**	0.88		**<0.001**
Ask how	0.458	**<0.001**		0.885	**<0.001**
Assistance	0.388	**<0.001**		0.575	**<0.001**

Note: Weighted Least Squares Means and Variance (WLSMV) adjusted estimator was used to model PPS data

### Regression analysis

The exploratory bivariate associations between the PPS and key covariates are located in Table 5. Higher total PPS scores (indicating a higher number of recent policing interactions) were observed among FSW who were homeless (β = 0.71, 95% CI: 0.04–1.37), conducted sex work daily (β = 1.32, 95% CI: 0.16–2.48), were arrested in the past 12 months (β = 1.44, 95% CI: 0.81–2.07) or injecting drugs in the past 3 months (β = 1.04, 95% CI: 0.52–1.57). Higher law enforcement patrol practices scores were associated with homelessness (β = 0.71, 95% CI: 0.16–1.26), daily sex work (β = 1.33, 95% CI: 0.37–2.28), arrest (β = 1.23, 95% CI: 0.71–1.75) and injecting drugs in the past 3 months (β = 1.27, 95% CI: 0.57–1.96). Higher police assistance patrol practices scores were associated with older age (β = 0.03, 95% CI: 0.01–0.04) and sex work for more than five years (β = 0.47, 95% CI: 0.13–0.81).

**Table 5:** Associations between PSS Score and sociodemographic, behavioral and police interaction characteristics of female sex workers (N = 250) in Baltimore, Maryland.

		Total PSS score		Law enforcement patrol practices score		Police assistance patrol practices score	
	N (col %)	β (95% CI)	p	β (95% CI)	p	β (95% CI)	p
Age: mean, SD	36 (9.0)	0.01 (-0.03–0.05)	0.597	-0.02 (-0.05–0.01)	0.31	0.03 (0.01–0.04)	<0.001*
Race/ethnicity							
Non-Hispanic White	166 (66.4)	REF		REF		REF	
Non-Hispanic Black	57 (22.8)	-0.50 (-1.28–0.28)	0.209	-0.63 (-1.28–0.01)	0.055	0.13 (-0.17–0.44)	0.40
Hispanic, Multiracial or Other	27 (10.8)	0.08 (-1.01–1.18)	0.883	0.24 (-0.66–1.14)	0.599	-0.16 (-0.59–0.27)	0.465
Highest level of education attained							
Did not complete high school	131 (52.4)	-0.13 (-0.78–0.52)	0.695	0.01 (-0.52–0.55)	0.957	-0.14 (-0.40–0.11)	0.262
Homeless, past 3 months	156 (62.4)	0.71 (0.04–1.37)	0.037*	0.71 (0.16–1.26)	0.011*	0.00 (-0.27–0.26)	0.979
Length in street-based sex work							
≤ 1 year	44 (17.6)	REF		REF		REF	
> 1 to 5 years	77 (30.8)	0.47 (-0.50–1.43)	0.342	0.30 (-0.50–1.10)	0.455	0.16 (-0.21–0.53)	0.392
> 5 years	129 (51.6)	0.79 (-0.10–1.68)	0.083	0.32 (-0.42–1.06)	0.398	0.47 (0.13–0.81)	0.008*
Frequency of street-based sex work							
Daily	165 (66.0)	1.32 (0.16–2.48)	0.026*	1.33 (0.37–2.28)	0.007*	0.00 (-0.47–0.46)	0.985
≤ Weekly	63 (25.2)	0.23 (-1.03–1.50)	0.716	0.40 (-0.64–1.44)	0.452	-0.16 (-0.67–0.34)	0.522
≤ Monthly	22 (8.8)	REF		REF		REF	
Arrested, past 12 months	116 (46.6)	1.44 (0.81–2.07)	<0.001*	1.23 (0.71–1.75)	<0.001*	0.20 (-0.05–0.46)	0.116
Injected drugs, past 3 months	177 (70.8)	1.04 (0.52–1.57)	<0.001*	0.91 (0.48–1.34)	<0.001*	0.14 (-0.07–0.36)	0.195
Sold or supplied drugs, past 3 months	58 (23.2)	-0.08 (-0.85–0.69)	0.843	0.09 (-0.55–0.73)	0.781	-0.17 (-0.47–0.13)	0.272

*p<0.05

## Discussion

This study is the first to put forward a measurement scale to help characterize and examine aggregate impacts of routine police patrol practices towards FSW and PWUD. It represents an innovative tool for measurement of a prevalent exposure that has been identified in the literature as a key structural determinant influencing risk behaviors and negative health outcomes experienced by both populations.[2,25,37] Using this tool, we observed a disproportionate burden of police interactions among the most vulnerable of FSW, those who were homeless or frequently injected drugs.

We observed two underlying factors characterizing routine patrol practices: practices consistent with ‘move along or stop and search’ (law enforcement patrol practices); and practices aligned with ‘community policing’ (police assistance patrol practices). Though the two were correlated, indicating the presence of an underlying construct, distinct associations between these factors and key covariates were observed in regression analysis. Law enforcement patrol practices included tactics commonly employed by the police force in the ‘war against drugs’ and previously to be associated with elevating HIV risk among PWID and eroding community-police relations in the U.S. context.[38–40] This factor is consistent with the theory of structural violence, reflecting an underlying inequality and power imbalance between the most vulnerable of FSW and the police. In contrast, police assistance patrol practices consisted of practices typically aligned with ‘community policing,’ which has been employed in several U.S. states and countries, including within harm reduction programs aimed at better alignment of law enforcement and public health objectives.[41] The distinction between the harms of routine policing and the positive aspects of community policing in communities that are often both ‘over-policed’ and ‘under-protected’ [42] was a strength of this scale.

Existing studies lack consistency and comparability with respect to identifying police behaviors and interactions that confer the most risk, and more robust tools are needed [25]. Further, there is a tendency to focus on overtly egregious acts which constitute abuses of power. While these are of clear public health and human rights importance, they are comparably rare and likely operate on the risk environment through a different mechanism. Among the small body of literature that does examine impacts of routine practices, studies have mainly focused on exposure to singular police practices, such as syringe confiscation or arrest[25]. Our results highlight the frequency of FSWs’ encounters with police using a more comprehensive list of items comprising routine policing practices (e.g., being asked to move along, ID and warrant checks); consideration these as separate items may lead to an underestimation of police interactions on the risk environment. Consistent with a shift towards understanding risk as a ‘complex system,’[9] having a scale that allows for a better exploration of the aggregate effect of seemingly innocuous day to day patrol police practices could be instructive in broadening our understanding of the significance of even routine policing as structural determinant of risk. The Patrol Practices Scale therefore provides a novel method to capture and assess the potentially insidious impact of previously unmeasured, aggregate routine police patrol practices on the health and safety of vulnerable populations.

In Baltimore City, a recent DOJ report documented the frequency of stops and concluded that they were rarely made in consideration of public safety goals, but instead were conducted with minimal supervision and led to constitutional violations (e.g., stops without reasonable suspicion).[43] FSWs’ encounters with police were infrequently driven by a custodial role characterized by offering help or assistance. These data quantitatively corroborate the qualitative literature[44,45] describing experiences of many similar populations in this context and elsewhere of feeling ‘over policed but under-protected’. [42] This form of over-surveillance by the police in the absence of any guardianship role is precisely the type of manifestation of ‘structural violence’ that needs to be captured; using this tool may help measure and discriminate between police encounters reflective of a community-based public safety approach, or structural violence characterized by zero-tolerance and intimidation.

This scale was developed based on ethnography and existing literature to achieve face validity, and our first scale also had a high internal consistency (α = 0.79). We conducted bivariate analyses to further explore the validity of our findings. Bivariate associations with key markers for FSW vulnerability (e.g., drug use, time on the street) highlight that the most structurally vulnerable FSW are disproportionately affected by routine policing practices. These also point to the relevance of this scale to the population of PWUD and PWID, given higher PPS scores were observed among those within the SAPPHIRE cohort who were characterized by high rates of illicit drug use and associated risks. These findings are illustrative of what Rhodes (2012) refers to as the ‘structuration of risk’ whereby certain social conditions (i.e., drug use and sex work) render some groups more likely to experience other forms of harm or discrimination; in this instance, that exposure is over-policing.[9] Consistent with qualitative data on the role of enforcement practices in the spatial control of urban landscapes,[46,47] higher markers of structural vulnerability such as being homeless and involved in daily sex work were also strongly associated with a higher PPS score. Conversely, no associations were detected between structural vulnerability indicators and exposure to ‘community policing.’

The study should be viewed in light of several limitations. The reliability of our second scale, police assistance, was modest (α = 0.53), which is likely a function of the low number of items included. More work is needed to explore the development of a police assistance sub-scale, with the inclusion of additional items; however, our data indicate that these items, whether assessed as two separate items or a more robust eventual scale, should be considered separately from the enforcement practices captured in the first scale. Second, for some items, past 3 month prevalence was lower than expected (e.g., confiscation of condoms and syringes); we encourage future users to use a longer timeframe (e.g., past 12 month) than used in the current study. The current scale assigns equal weights to each PPS item; this assumption should be tested in future research. Some items were collapsed during analysis and future work should use the revised version of the scale included as Fig 1 in this paper. It is also important to note the racial demographic of the population in which this scale was developed. While Baltimore is a majority (60%) African American city, the sample of cis-gendered participants in the study (i.e., street-based FSW) was predominantly white (66%). The relative underrepresentation of African American FSW in the cis-gendered SAPPHIRE cohort may be due to fundamentally different geographic and structural patterns of finding and engaging with clients and researchers, some of which may be driven by differences in police interactions; for example, due to fear of police harassment or arrest, African American FSW may be: (1) more likely to engage in sex work in an online/private setting rather than at street-based locations; (2) disproportionately incarcerated; or (3) prefer more private interview locations. While our findings in the SAPPHIRE cohort indicate that FSW in all racial categories are affected by policing, perhaps due to the marginalization of street-based sex workers overall, it is important that the reliability and validity of this scale be tested among different populations with greater representation of racial minority women. We have included a modified scale as Fig 1 in this article with survey items that have been collapsed based on our study findings. Finally, although the majority of the SAPPHIRE cohort were also PWUD, the scale reliability and validity were not examined specifically with this population in mind, and further research is warranted.

## Conclusions

This study provides a novel and relevant scale for use across two overlapping street-based populations for whom policing represents an important structural determinant of risk. In particular, it addresses the ongoing need to quantitatively operationalize the evolving conceptual theories of police’s contribution to FSW and PWIDs’ experience of structural violence.[9] The PPS can be applied to other street-based populations who experience high levels of day to day police exposure, in particular homeless populations, transgender women and racial minorities.[48,49]. Among FSW and PWUD population research, the PPS may provide a useful scale to capture routine police patrol practices and how they can mediate risk of key outcomes including HIV/STI incidence and overdose. This could provide an important evidence base from which public health researchers can work with police departments on the design and implementation of interventions that target these practices. It should be noted that for female transgender sex workers in this study cohort initial application of the PPS points to a lower internal consistency of the scale, suggesting that formative research will be critical to capturing the effect of day to day policing on sub-populations health-related outcomes. The public health and human rights imperative remains decriminalization of sex work[7], accompanied by ongoing efforts to re-orientate policing approaches to align with public health and harm reduction. However, both require a strong evidence base demonstrating the wide range of potential consequences of conducting business as usual policing; a greater focus on these practices, alongside more blatant and egregious events, thereby providing an important point of evidential leverage with police departments. Informing the development of structural interventions that incorporate police-public health partnerships is crucial to addressing the underlying risk of these vulnerable populations, and may be best achieved through understanding and addressing the most ubiquitous of police patrol practices first.

## Supporting information

S1 Table Police interactions measured using the Patrol Practices Scale (PPS) items among transgender female sex workers (N = 62) in Baltimore, Maryland. (DOCX)

S2 Table Internal consistency measures for using a novel Police Practices Scale (PPS) in a population of transgender female sex workers (N = 62) in Baltimore City. (DOCX)
