# Supplementary material for: The development of the Police Practices Scale: Understanding policing approaches towards street-based female sex workers in a U.S. City
Source: PLoS One. 2020 Jan 24;15(1):e0227809. doi: 10.1371/journal.pone.0227809 (PMC6980607; doi:10.1371/journal.pone.0227809)
Supplement: S1 Table — (DOCX) [file pone.0227809.s001.docx]

**S1 Table. Police interactions measured using the Patrol Practices Scale (PPS) items among transgender female sex workers (N=62) in Baltimore, Maryland**

| Item  no. |  | Never in past 3 months | Once a month or less | More than once a month | Once a week | More than once a week | Daily |
| --- | --- | --- | --- | --- | --- | --- | --- |
| 1 | Asked you to move on from a specific stroll or other public space. | 22 (35.5) | 16 (25.8) | 8 (12.9) | 6 (9.7) | 8 (12/9) | 2 (3.2) |
| 2 | Asked how you’re doing or inquired about your wellbeing. | 39 (62.9) | 11 (17.7) | 4 (6.5) | 5 (8.1) | 1 (1.6) | 2 (3.2) |
| 3 | Asked to see a form of I.D. | 26 (41.9) | 20 (32.3) | 7 (11.3) | 2 (3.2) | 4 (6.5) | 3 (4.8) |
| 4 | Run a warrant check. | 36 (58.1) | 16 (25.8) | 3 (4.8) | 2 (3.2) | 4 (6.5) | 1 (1.6) |
| 5 | Conducted a search of your person and property (e.g. patted you down, looked through your bag). | 49 (79.0) | 10 (16.1) | 2 (3.2) |  |  | 1 (1.6) |
| 6 | Confiscated or destroyed your condoms. | 58 (93.6) | 2 (3.2) | - | - | - | 1 (1.6) |
| 7 | Confiscated drugs from you. | 60 (96.8) | - | 1 (1.6) | - | 1 (1.6) | - |
| 8 | Confiscated syringes or drug paraphernalia from you. | 61 (98.4) | 1 (1.61) | - | - | - | - |
| 9 | Helped you out without expecting anything in return e.g. bought you food or drink, or given you a ride to services. | 51 (82.3) | 10 (16.1) | - | - | - | 1 (1.6) |
| 10 | Referred you to health or social services e.g. drug or alcohol treatment, or a violence shelter. | 61 (100.0) | - | - | - | - | - |
|  |  |  |  |  |  |  |  |
