# Supplementary material for: The development of the Police Practices Scale: Understanding policing approaches towards street-based female sex workers in a U.S. City
Source: PLoS One. 2020 Jan 24;15(1):e0227809. doi: 10.1371/journal.pone.0227809 (PMC6980607; doi:10.1371/journal.pone.0227809)
Supplement: S2 Table — (DOCX) [file pone.0227809.s002.docx]

**S2 Table: Internal consistency measures for using a novel Police Practices Scale (PPS) in a population of transgender female sex workers (N=62) in Baltimore City**

| *Cronbach’s α* | |  |  |  |  |
| --- | --- | --- | --- | --- | --- |
|  | Full scale (9-items; excluding item 6) |  |  |  | 0.65 |
|  | Final scale (6-items; collapsed items 3 & 4; items 7 & 8; items 9 & 10) | |  |  | 0.58 |
|  | Law enforcement patrol practices (4-items) |  |  |  | 0.61 |
|  | Police assistance patrol practices (2-items) |  |  |  | 0.57 |
